# Primary pulmonary angiosarcoma mimicking acute interstitial pneumonia – a diagnostic challenge: A case report

**DOI:** 10.1097/MD.0000000000042106

**Published:** 2025-04-25

**Authors:** Magdalena Onyszczuk

**Affiliations:** a Department of Pathomorphology, Faculty of Medical Sciences in Zabrze, Medical University of Silesia, Katowice, Poland.

**Keywords:** angiosarcoma, autopsy, case report, lung

## Abstract

**Rationale:**

Angiosarcoma is a malignant endothelial cell tumor of vascular or lymphatic origin and accounts for approximately 2% of all soft tissue neoplasms. Primary pulmonary angiosarcoma (PPA) is extremely rare, with <30 cases reported in the literature. Moreover, because of non clinical manifestation and low positive diagnostic rate of traditional, noninvasive or minimally invasive diagnostic methods, the early diagnosis of this neoplasm is really challenging.

**Patient concerns:**

A 73-year-old woman initially presented with anemia and hemoptysis, which has occurred over a period of 4 weeks before admission. Subsequently, the patient experienced progressive weakness, anxiety attacks, and loss of appetite.

**Diagnoses:**

The bronchoscopy and chest X-ray failed to detect the cause of the symptoms. The patient was initially treated for acute interstitial pneumonia. Subsequent video-assisted thoracic surgery with parietal pleura sampling identified the presence of neoplastic tissue of unknown primary origin.

**Interventions and outcomes:**

Transfusion of red blood cell concentrate and high-flow oxygen therapy were administered. In view of poor performance status and unfavorable prognosis, a decision was made not to escalate the therapy. The patient died 21 days after admission. An autopsy was ordered to determine the exact cause of death and revealed multiple nodular lesions scattered in both lung parenchyma and on the pleural surface. The final diagnosis of poorly differentiated epithelioid PPA was made on the basis of histopathological and immunohistochemical postmortem examination.

**Lessons:**

Presented case cautions that PPA should be considered in patient with hemoptysis, negative bronchoscopy and non-characteristic radiological findings. When the disease is suspected, biopsy and microscopic examination should be implemented to confirm the diagnosis as soon as possible and apply timely treatment. Moreover, due to the small number of PPA cases there is no standard diagnostic and treatment protocol available. Thus, further investigations and sharing experiences, may aid better understanding of this malignancy and lead to improvement in patients’ survival.

## 1. Introduction

Angiosarcoma is a malignant tumor of endothelial origin, arising from vascular or lymphatic tissue, and accounts for approximately 2% of all soft tissue neoplasms. Pulmonary angiosarcoma can be categorized into primary and metastatic forms. In most cases, pulmonary angiosarcoma represents metastasis from other primary sites, primarily the skin and subcutaneous tissues of the head and neck.^[1,2]^ Primary pulmonary angiosarcoma (PPA) is extremely rare, with fewer than 30 cases reported in the English literature.^[3,4]^ Early diagnosis of PPA is uncommon due to its rarity and low index of suspicion. This study presents a case of PPA mimicking acute interstitial pneumonia from both imaging and clinical perspectives. Given the rarity of this malignancy, our understanding of PPA and the available literature on the topic are limited.

## 2. Case presentation

**Figure 1.**
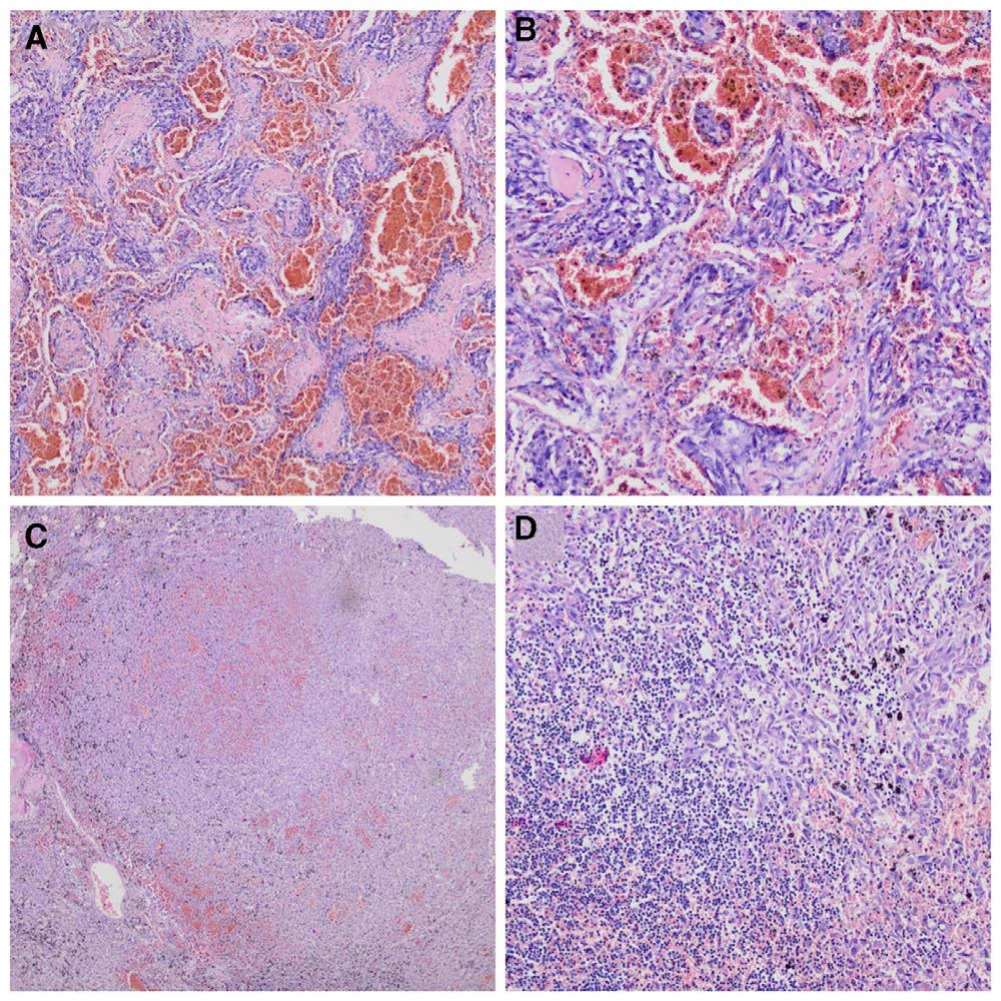
Histopathological findings of primary pulmonary angiosarcoma. (A) Microscopic image of one of the pulmonary tumor showing poorly differentiated proliferation of atypical, highly pleomorphic, malignant endothelial cells. The architecture is chaotic, without definitive vasoformation, accompanied by necrosis and intratumoral hemorrhage (hematoxylin & eosin [HE] staining, magnification ×100). (B) On microscopy, tumor cells are plump, mitotically active, have a large nuclear-cytoplasmic ratio, dark-colored nuclei and atypia (HE staining, magnification ×200). (C, D) Metastatic spread of the primary pulmonary angiosarcoma to the enlarged mediastinal lymph node with anthracosis (HE staining, magnification ×40 and ×100).

**Figure 2.**
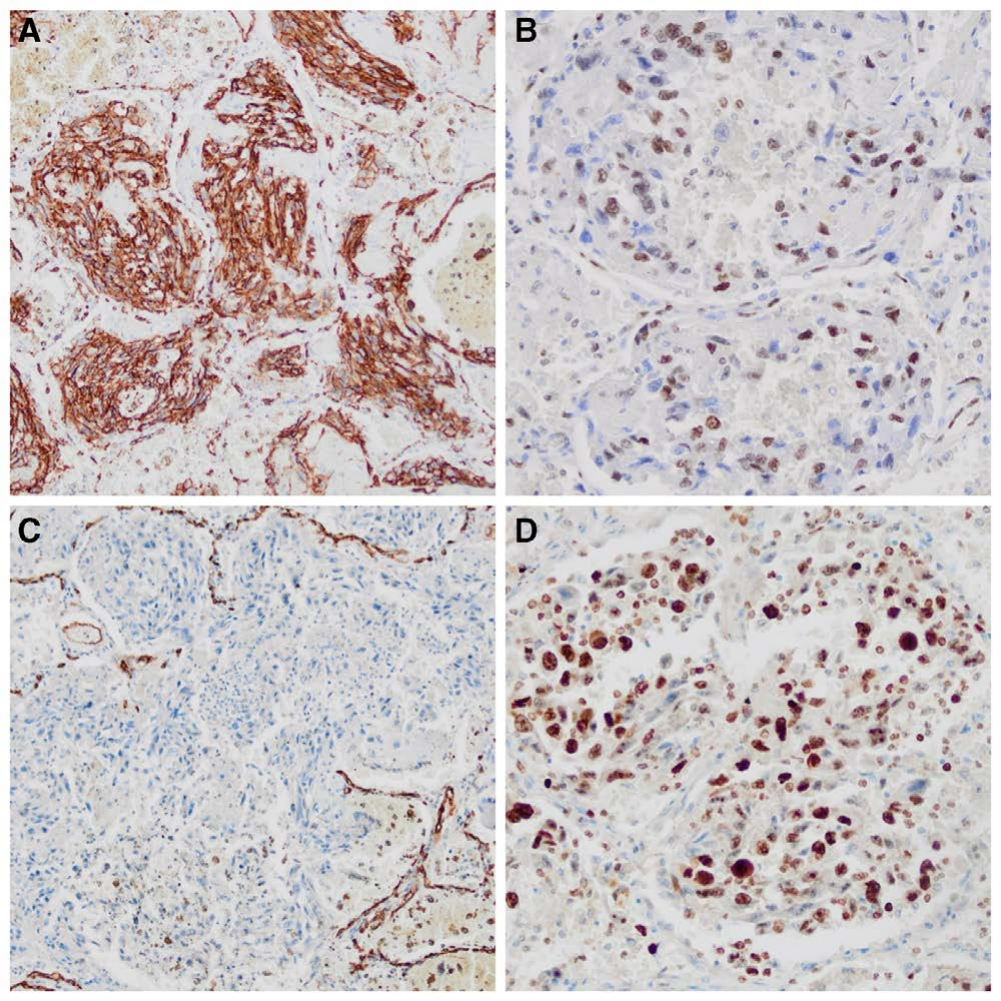
Immunohistochemical findings of primary pulmonary angiosarcoma. (A) Immunohistochemical examination with marker CD31 showing strong and diffuse membranous positivity in tumor cells (CD31 staining, magnification ×100). (B) Immunohistochemical examination with ERG antibody showing nuclear positivity in most tumor cells (ERG staining, magnification ×200). (C) Immunohistochemical negativity of the neoplastic cells for vascular marker CD34; note internal control in normal endothelium (CD34 staining, magnification ×100). (D) Ki-67 immunostaining showing high cell proliferative activity within the tumor (Ki-67 staining, magnification ×400). ERG = V-ets erythroblastosis virus E26 oncogene homolog.

A 73-year-old woman with a history of hypothyroidism, hypertension, and ischemic heart disease presented with anemia (hemoglobin 9.7 g/dL) and hemoptysis, which had occurred over a 4-week period before admission. At that time, she underwent bronchoscopy with bronchoalveolar lavage (BAL). The bleeding site was not localized. The BAL fluid was sent for culture and multiplex PCR testing to detect pathogens causing lower respiratory tract infections (bacteria, viruses, fungi, and atypical bacteria). Additionally, a BAL sample was submitted for cytological evaluation. However, all performed tests were negative for neoplastic cells and pathogens, including *Mycobacterium tuberculosis*. Based on the microbiological and pathological test results, along with the clinical manifestations, acute interstitial pneumonia was diagnosed, and treatment with prednisone and ceftriaxone was administered for 7 days. After being discharged from the hospital, the patient experienced progressive weakness, anxiety attacks, and loss of appetite, while denying any symptoms of gastrointestinal bleeding. Due to a lack of clinical improvement, progressive weakness, anemia (hemoglobin 7.3 g/dL), and persistent hemoptysis, a chest X-ray was performed and revealed fluid in the right pleural cavity and multifocal confluent densities in both lungs interpreted as inflammatory changes. The patient underwent right-sided video-assisted thoracoscopic surgery (VATS) for hemothorax evacuation, talc pleurodesis, and parietal pleura sampling. Histological examination of the biopsy specimen showed neoplastic tissue of unknown primary origin. Due to significant anemia (hemoglobin 5.4 g/dL) and progressive respiratory failure, a transfusion of red blood cell concentrate and high-flow oxygen therapy were administered. Given the serious clinical condition, poor performance status, and unfavorable prognosis, the treating team, in consultation with the patient, decided not to escalate therapy. The patient died 21 days after admission. An autopsy was ordered to determine the exact cause of death, revealing bilateral hemothorax, atelectasis, and multiple nodular cream-brown solid lesions up to 1.5 cm in diameter scattered throughout both lung parenchyma and on the pleural surface. Enlarged mediastinal lymph nodes up to 2 cm in size were also noted. No tumor was found in other organs. Histological examination of the lung tissue revealed poorly differentiated proliferation of atypical, highly pleomorphic, epithelioid cells with chaotic architecture, lacking definitive vasoformation, and accompanied by necrosis and intratumoral hemorrhage. The tumor cells were plump, mitotically active, had a large nuclear-cytoplasmic ratio, dark-colored nuclei, and abundant eosinophilic cytoplasm. Neoplastic cells were also detected in the lymph nodes (Fig. 1). Immunohistochemical testing showed that the neoplastic cells stained positive for ERG (V-ets erythroblastosis virus E26 oncogene homolog) and CD31 but negative for CD34, cytokeratin (CK), smooth muscle actin, STAT6, S100, HMB-45, and CD30 (Fig. 2). Therefore, both histology and immunohistochemistry were consistent with disseminated poorly differentiated epithelioid PPA with nodal metastases.

## 3. Discussion

Pulmonary angiosarcoma is a rare type of neoplasm derived from vascular endothelial cells. PPA accounts for only 9.3% of all lung angiosarcomas.^[1]^ The mean age of onset is 54.7 years, with occurrences being more common in men than in women.^[1,4]^ The clinical manifestations of PPA are nonspecific, with symptoms similar to those of lung cancer, such as hemoptysis or hemosputum, cough, dyspnea, chest pain, weight loss, fever, and fatigue, as seen in the present case.^[1,2]^ However, up to 20% of patients may be asymptomatic, and the disease is detected incidentally or during autopsy.^[1–5]^

Early diagnosis of PPA is uncommon due to the ill-defined respiratory manifestations and the consequent low index of suspicion. Imaging plays an essential role in detecting malignancy. Unfortunately, chest X-ray often fails to reveal subtle changes associated with this neoplasm, as confirmed by our case description.^[1]^ Bronchoscopy also demonstrates a low positive diagnostic rate. Several factors contribute to the infrequent diagnosis through this method, including the limited presence of tumors in the bronchi and the challenging recognition of the unusual pathological characteristics of PPA from small biopsy samples.^[4]^ In our patient, bronchoscopy did not reveal any intrabronchial space-occupying lesions, which pointed to an infectious nature and led to the initial misdiagnosis of interstitial pneumonia. On computed tomography scans, PPA may manifest as solitary or multifocal nodular lesions, with or without ground-glass or solid changes, which can contribute to misdiagnoses such as diffuse pulmonary hemorrhage, tuberculosis, or pneumonia.^[1,2,6–8]^ Since PPA can spread to different organs by the time of presentation, positron emission tomography imaging can also provide valuable information for staging due to its sensitivity to metastatic disease, as well as follow-up of patients with PPA.^[2,9,10]^ However, none of the imaging methods can definitively differentiate PPA from other pulmonary malignancies; therefore, in cases of high clinical suspicion, medical thoracoscopy/VATS, or an open lung biopsy are the most effective diagnostic approaches.^[4]^ In the case presented here, VATS with pleura sampling confirmed its utility.

The final diagnosis is based on histopathological and immunohistochemical findings.^[1,4]^ On microscopic examination, angiosarcomas typically exhibit ill-defined margins and a wide morphologic appearance, ranging from cytologically bland lesions with well-formed vascular channels lined by minimally atypical spindle cells (more rarely) to solid sheets of high-grade epithelioid or spindle cells without definitive vasoformation. Vasoformative areas are composed of irregularly branching, anastomosing vascular channels lined by atypical endothelial cells, with variable multilayering, intraluminal budding, hobnailing, or papillary-like projections. Solid areas typically comprise cellular sheets of plump, pleomorphic, and mitotically active cells with abundant eosinophilic to amphophilic cytoplasm, large vesicular nuclei, and prominent nucleoli. There may be associated intratumoral blood lakes or extensive hemorrhage, with organizing hematoma obscuring much of the neoplasm. Combined with heterogeneous morphological features, this makes the histological identification of angiosarcoma a significant challenge. Epithelioid angiosarcomas typically exhibit a solid architecture with a diffuse, sheet-like pattern of large, atypical epithelioid or polygonal cells, characterized by ovoid vesicular nuclei, prominent large central nucleoli, and abundant cytoplasm, which is consistent with the autopsy results of our patient.^[11,12]^

PPA typically expresses endothelial markers, such as ERG, factor VIII-related antigen, CD31, CD34, VEGF, and FLI1. Currently, ERG is one of the best available markers of endothelial differentiation, confirming the vascular origin of the tumor. CD34 is a less sensitive and specific endothelial marker than CD31, and relying solely on this marker for vascular differentiation in an immunohistochemical panel is insufficient and could lead to diagnostic delays or errors. In a subset of angiosarcoma cases, particularly in epithelioid subtypes, tumor cells may express cytokeratin (CK) and epithelial membrane antigen, which can lead to misdiagnosis as carcinoma. CD30 may also be expressed in a subset of epithelioid angiosarcomas, while the lymphatic marker D2-40 is variably expressed, suggesting focal lymphatic differentiation in the tumor.^[1–4,11,12]^ Positive ERG and CD31 staining, combined with histopathological findings from autopsy tissue, confirmed our final diagnosis. However, it is important to note that although the literature supports CD34 staining as one of the most sensitive markers for angiosarcoma, it can be negative, as it was in our case. Therefore, the final diagnosis should not rely solely on the results of a single immunohistochemical marker of vascular differentiation.

Due to the rarity of this neoplasm, no unified therapy recommendations have been established to date, especially for PPA. Generally, treatment options depend on the stage of the disease. Surgery, radiotherapy, chemotherapy, immunotherapy, or a combination of these modalities have all been attempted; however, none have demonstrated consistent effectiveness, particularly in cases of metastatic disease.^[1–4,6,7,10,13]^ Surgical resection is the main treatment modality and should be performed as early as possible for locally confined tumors.^[2,4,12,14]^ Unfortunately, neoplasms are often diagnosed at a late stage and may be inoperable at that time, as in our patient. For patients with small tumors (especially those <3 cm) or those who are unable to undergo surgery, local ablative treatment may be an alternative option. Additionally, vascular embolization may be performed prior to surgery, particularly in patients with large tumors and significant hemorrhagic symptoms.^[3,15]^ After surgery, adjuvant radiotherapy and aminolevulinic acid-photodynamic therapy serve as complementary treatments due to the tumor radiosensitivity.^[3,16,17]^ Given the risk of metastasis, anthracycline-based chemotherapy is also recommended.^[3,18]^ Chemotherapy is the primary treatment for advanced-stage disease, with doxorubicin, with or without ifosfamide, being the most commonly prescribed regimen. The combination of gemcitabine and docetaxel has also proven to be highly effective, as it has been reported to induce a complete radiologic response.^[1–4,10]^ Nonetheless, chemotherapy is primarily used as palliative treatment for angiosarcoma when a significant response is not observed.^[3,6]^ Another promising approach involves vascular-targeted therapies; research indicates that antiangiogenic agents like bevacizumab and sorafenib can be effective in managing angiosarcomas.^[3,18]^ Additional potential treatments for angiosarcoma, such as vascular-disrupting agents (ASA404) and immune modulators (recombinant interleukin-2 or interferon-α), may also extend progression-free survival.^[1–3,13,19,20]^ New findings suggest that immune checkpoint inhibitors could be effective for angiosarcoma, but further investigations are necessary.^[3,21]^ Unfortunately, because PPA is an exceedingly rare sarcoma, identifying the most appropriate treatments for patients remains a considerable challenge.

The prognosis for PPA is extremely unfavorable, with 5-year survival rates ranging from 16% to 56%.^[3,22]^ Patients with multiple lesions have a poorer prognosis (median survival time of 2 months) compared to those with a solitary tumor (median survival time of 7 months).^[1,4]^ In the present case, the disease progressed rapidly and exhibited aggressive characteristics, presenting with advanced inoperable lesions that ultimately led to death within <2 months. Consequently, early detection and diagnosis are essential.

The main limitations of this study relate to its retrospective design and the single-case nature, which limits the generalizability of the findings. Furthermore, the absence of long-term follow-up significantly reduces the reliability of this research.

## 4. Conclusion

In summary, we report a very rare case of PPA misdiagnosed as acute interstitial pneumonia, which was accurately diagnosed after autopsy using histopathology and immunohistochemical biomarkers. This neoplasm poses a diagnostic challenge for both clinicians and pathologists due to the lack of specific clinical manifestations. This case highlights the need to consider PPA in patients presenting with hemoptysis, negative bronchoscopy results, and nonspecific radiological findings. When the disease is suspected, a biopsy and microscopic examination should be performed promptly to confirm the diagnosis and facilitate timely treatment.

## Author contributions

**Conceptualization:** Magdalena Onyszczuk.

**Data curation:** Magdalena Onyszczuk.

**Formal analysis:** Magdalena Onyszczuk.

**Funding acquisition:** Magdalena Onyszczuk.

**Investigation:** Magdalena Onyszczuk.

**Resources:** Magdalena Onyszczuk.

**Supervision:** Magdalena Onyszczuk.

**Visualization:** Magdalena Onyszczuk.

**Writing – original draft:** Magdalena Onyszczuk.

**Writing – review & editing:** Magdalena Onyszczuk.
